# Policy challenges from the "White" Senate inquiry into workplace-related health impacts of toxic dusts and nanoparticles

**DOI:** 10.1186/1743-8462-3-7

**Published:** 2006-06-17

**Authors:** Thomas A Faunce, Haydn Walters, Trevor Williams, David Bryant, Martin Jennings, Bill Musk

**Affiliations:** 1 Medical School and College of Law Australian National University, Canberra, Australia; 2 Royal Hobart Hospital, Clinical School University of Tasmania, Hobart, Australia; 3 Department of Allergy, Immunology & Respiratory Medicine, The Alfred Hospital, Melbourne, Australia; 4 Department of Medicine St Vincent's Clinical School and University of NSW, Sydney, Australia; 5 Past President Australian Institute of Occupational Hygenists, Australia; 6 Faculty of Public Health and Medicine University of Western Australia, Perth, Australia

## Abstract

On 22 June 2005 the Senate of the Commonwealth of Australia voted to establish an inquiry into workplace harm related to toxic dust and emerging technologies (including nanoparticles). The inquiry became known as the "White" Inquiry after Mr Richard White, a financially uncompensated sufferer of industrial sandblasting-induced lung disease who was instrumental in its establishment. The "White" Inquiry delivered its final report and recommendations on 31 May 2006. This paper examines whether these recommendations and their implementation may provide a unique opportunity not only to modernize relevant monitoring standards and processes, but related compensation systems for disease associated with workplace-related exposure to toxic dusts. It critically analyzes the likely role of the new Australian Safety and Compensation Council (ASCC) in this area. It also considers whether recommendations related to potential workplace related harm from exposure to nanoparticles could commence a major shift in Australian healthcare regulation.

## Background

On 22 June 2005 the Senate of the Commonwealth of Australia voted to establish an inquiry into workplace harm related to toxic dust and emerging technologies (including nanoparticles). The inquiry has become known as the "White" Inquiry after Mr Richard White, a financially uncompensated sufferer of industrial sandblasting-induced lung disease whose sentinel case and advocacy through the Australian Sandblasting Diseases Coalition (ASDC) was instrumental in its establishment. Its final report was delivered on 31 May 2006 [1].

Numerous government inquiries into toxic dust exposure in Australia have been held since the turn of the century. They have focused, for example, on worker health problems associated with the Western Australia and Victorian goldmines and NSW and Queensland coalmines, as well as the Sydney sandstone industries (through the NSW Silicosis Board (now the Dust Diseases Board)) [2]. In 1993, the National Occupational Health and Safety Commission reviewed the occupational exposure standard for crystalline silica (silicon dioxide (SiO2) chiefly from abrasive blasting, excavating, quarrying or tunneling quartz and granite, but including the aggregates, sand, mortar, concrete and stone)[3].

There are, however, many reasons to consider that the level of disease associated with workplace disease from toxic dust continues to be much greater than revealed by such inquiries or reviews. First, the long latency period of the relevant diseases and the insidious nature of their acquisition, mean that the actual incidence and prevalence of workplace injury from toxic dusts remains largely unknown. Second, it remains unclear what exposure levels (mg/m3) effectively minimize injury[4]. Occupational exposure limits for crystalline silica, for example, are almost constantly under review worldwide because of the large numbers of exposed people[5]. The position is further complicated because accurate exposure monitoring and hence enforcement of standards, is generally more difficult at lower exposure levels[6].

Third, associated cigarette smoking often complicates causation analysis in workers regularly exposed to toxic dusts[7]. This is particularly true where the disease manifestation is lung cancer [8] and many potentially carcinogenic compounds are used in the workplace[9]. Fourth, many workers suffering disease associated with exposure to toxic dusts during employment will not seek, let alone receive compensation[10]. Fifth, the infrastructure for successful implementation of national standards (including the number of occupational hygenists in government employment) has been eroded and few if any Australian companies have been prosecuted for exposing workers to the risk of dust-related disease [2].

A sentinel case highlighting such problems was provided by Mr Richard White.

## Richard White's silicosis: a sentinel case for policy change

As a teenager, Mr Richard White was employed, between 1971 and 1974 in the Northern Territory, to sandblast clean barges and fuel tanks, then spray them with an epoxy resin containing many of the carcinogens listed above. He did this without being issued respiratory protection. In the 1990's, Mr White began to notice severe dyspnoea, fatigue, paroxysmal coughing and sputum production, particularly when playing with his children. He suffered repeated chest infections and initiated a compensation claim in the Northern Territory Supreme Court. This alleged that as a result of this employment, he had developed silicosis and/or emphysema and/or chronic air flow limitation[11]. He was initially met with legal defence arguments about the liability of subsidiary companies, similar to those raised in the James Hardie asbestos litigation[12]. It is a matter of record that Mr White lost the first instance trial, the subsequent appeal to the Supreme Court of the Northern Territory and an appeal to the High Court of Australia. His history of mild smoking (reported as < 5 pack years) was a significant factor in the case against him, as was a defence-proffered allegation of asthma, for which there was no known medical history [13].

The histopathology of Mr White's subsequent open lung biopsy, revealed, as well as some adjacent emphysema:

"*Scant brightly birefringent, needle-shaped crystalline material, consistent with inhaled silicate crystals, are noted within these macrophages. In addition, similar material is noted in macrophages within the lymphoid aggregates of the dust sumps, with a miniscule amount of associated interstitial fibrosis*".

These results strongly suggested Mr White had industrially-related silica injury to his lungs, in the form of interstitial fibrosis, small airways disease and emphysema, giving rise to mainly fixed airflow obstruction. Mr White thereafter placed a newspaper advertisement. It requested people to contact him who knew or suspected they had acquired lung or other disease through working for companies that used sandblasting techniques. By Christmas 2004, Mr White had obtained almost a thousand names[13].

Many of these people claimed to have experienced symptoms consistent with debilitating lung diseases or cancer related to workplace exposure to toxic dusts. Very few had received or sought any compensation. Given the relatively informal way in which the list was been prepared, Mr White began to believe that many other Australians has suffered potentially harmful exposure to toxic workplace dust, without ever seeking more specific diagnosis or financial compensation[13].

Mr White and the first author commenced lobbying for a Senate Inquiry into the workplace risks of toxic dusts in Australia as the best way to initiate a change in health policy in this area. Late in June 2005 Senators Lyn Allison (Dem.) and Gary Humphries (Lib.) successfully obtained the majority required to initiate that Inquiry. Its terms of reference were expanded to include the potential of emerging technologies, including nanoparticles, to result in workplace related harm.[14] The recommendations of the "White" Senate Inquiry urge the new Australian Safety and Compensation Council (ASCC) toward some far-reaching changes in the system of prevention and monitoring of dust-related disease in Australia.

## Policy recommendations for general workplace toxic dust exposure

Chronic Obstructive Pulmonary Disease ("COPD") is currently the fourth leading cause of death worldwide and the only chronic lung disease whose incidence in the developed world continues to increase. An analysis of 10 large scale studies (taking into account tobacco smoking status) in the US, France, Spain, Norway, the Netherlands, Italy, China and New Zealand, indicates that approximately 15% of the burden of illness from COPD arises from workplace exposure to toxic dust[15]. This is unlikely to be a "worst-case" estimate, rather, the studies reveal that much COPD is undiagnosed[16]. In Australia, recent epidemiological studies in middle aged Melbourne residents have shown that a relatively significant amount of COPD is not related to smoking, and industrial exposures are a significant contribution[17]. There is now good pathological evidence in both humans and animals of the capacity of crystalline silica to cause emphysema[18]. Australian healthcare policy and related monitoring and compensation systems appear, however, not to have fully appreciated the regulatory significance of this advance in causal understanding.

The British Coal litigation, on the other hand, was a watershed in the development of clinical and legal theories about causative relationships between industrial dust exposure and COPD. One of its major conclusions was that disability in a toxic dust-exposed cigarette smoker should not be regarded for compensation purposes as if it was entirely due to one cause or the other. Rather the courts in that nation decided they should attempt to estimate, as far as possible, the contribution of each such cause and then award compensation proportionally[19]. A related recommendation, posing an obvious challenge to Australian healthcare policy, was that compensation should prima facie be paid to any worker with COPD who has worked underground for 20 years, even in the absence of pneumoconiosis on chest x-ray[20].

The "White" Senate Inquiry recommended that the ASCC review the National Data Action Plan, to increase the availability of relevant data (Rec. 1). It required the ASCC to extend the Surveillance of Australian Work-Based Respiratory Events (SABRE) program Australia-wide, to provide mandatory reporting of dust-related disease (Rec. 2). Most importantly, it required the ASCC in conjunction with the Heads of Workplace Safety Authorities consider mechanisms to increase the number of occupational hygienists being trained and employed by regulators (Rec.8). The Minister for Employment and Workplace Relations is asked to raise with the Workplace Relations Ministers' Council the need to enact nationally consistent standards for identification, assessment and compensation for sufferers and their families based on at least the standard on the NSW *Workers Compensation *(*Dust Diseases Act*) *1942*, as well as removing restrictive statutes of limitation (Rec. 7, 9, 10 & 11)[1].

These recommendations, particularly the last, provide strong policy opportunities for an Attorney General with a firm interest in unifying areas of legal regulation in Australia and a Federal government willing to display its genuine concern for protecting worker safety in a period of considerable upheaval in workplace relations. They set a challenge for the ASCC that should be called to account for the steps taken toward their implementation by March 2007. State governments displaying a lack of willingness to become involved in this long overdue regulatory rationalization should also have their credentials on workplace safety called into question.

## Policy challenges from workplace exposure to nanotechnology

Nanoparticles are very small. A nanometer is one-billionth of a metre (a human hair is 80,000 nm wide)[21]. Nanoparticles particularly used in transparent sunscreens and cosmetics, "smart" surveillance equipment, fertilizers and packaging, nutritionally enhanced foods, long-lasting paints and as industrial catalysts. They may also arise from thermal spraying, metal production and refining, welding, soldering and high speed metal grinding[22]. Medical nanotechnology involves the development of drug/invasive therapeutic device products controllable at atomic, molecular or macromolecular levels of approximately 1–100 nanometers. It is a rapidly expanding area of research globally with revolutionary implications for disease detection and analysis,[23] drug delivery,[24] and reconstructive,[25] neurological[26] and cardiac surgery[27]. Australian companies are already working, for example, on nanotechnology-based sunscreen, anticoagulant and drug delivery products[28].

Nanoparticles present unique health risks, being extremely reactive whilst readily penetrating mucosal membranes, entering blood vessels and impacting on the coagulation system. There are currently no effective methods to measure and assess exposure risks to nanoparticles in patients or healthcare workers. Nanoparticle exposure limits do not exist and manufacturers currently have no obligation to publish details of the safety checks imposed on their nanoproducts. A long latency period for disease from exposure to nanoparticles and the insidious symptom development, mean causation will be difficult to legally prove and compensation difficult to obtain.[29] Sketchy nanomedicine safety and toxicity profiles thus may create major policy challenges, not only for Australian Therapeutic Goods Administration (TGA) marketing approvals, but for Pharmaceutical Benefits Advisory Committee (PBAC) and Medical Services Advisory Committee (MSAC) cost-effectiveness evaluations, as well as the horizon scanning program of the Health Policy Advisory Committee on Technology (HealthPACT). Interdepartmental meetings, industry consultations and discussions on international harmonisation, have occurred on the health risks of nanotechnology generally (and will continue under the interdepartmental committee of the National Nanotechnology Strategy Taskforce ("NNST") within the Department of Industry, Tourism and Resources)[30]. A 2005 report to the Prime Minister's Science Engineering and Innovation Council warned: "The early introduction and explanation of regulation reduces the risk that public concern will prevent acceptance of nanotechnology. Industry also tends to prefer certainty in regulation"[31].

The "White" Senate Inquiry recommended that the National Nanotechnology Strategy be finalized as a matter of priority (Rec. 12). It required establishment of a widely consulting working party on nanotechnology regulation, comprising representatives of the Therapeutic Goods Administration, the National Industrial Chemicals Notification and Assessment Scheme (NICNAS), and the ASCC. This is to consider (with consideration of international models) the appropriateness of existing regulations, how gaps and uncertainties in that regulatory framework can be addressed and risk management incorporated, possible reassessment of safety and whether a permanent nanotechnology regulatory body needs to be established. (Rec. 12, 13 and 14)[1].

These recommendations present an opportunity for Australia to develop an innovative and practical regulatory framework that will not only facilitate the development of an important industry sector, but ensure the safety of workers and those members of the public associated with its products. Implementing these policy recommendations becomes a matter of national urgency, given the burgeoning level of research already taking place in this area in Australia.

## Conclusion. Policy challenges for the ASCC

The new ASCC, replacing the National Occupational Health and Safety Commission (NOHSC), first met on 20 October 2005. The ASCC comprises representatives from Federal, State and Territory Governments, the Australian Council of Trade Unions (ACTU) and the Australian Chamber of Commerce and Industry (ACCI). One of its main aims is to provide policy advice to the Workplace Relations Minister's Council on OHS and workers' compensation arrangements. A related purpose is to deliver nationally consistent frameworks providing leadership and coordination to prevent workplace death, injury and disease through two working groups: an OHS Working Group, and a Workers Compensation Working Group[32].

There are many core policy challenges that the ASCC will have to address in implementing the recommendations of the "White" Senate Inquiry. The first is that the crucial problem of workplace-related disease from toxic dusts, both world-wide and in Australia, has not been one of creation of standards, but of their implementation[33]. Standards, for example, prohibiting abrasive sand blasting and providing recommended exposure limits for respirable crystalline silica, as well as respiratory protection, worker education and regular medical examinations have been in place in Australia since the late 1960's and early 1970's[34]. Since that time, funding has been reduced for inspectors and insufficient attention paid to increasing their powers. It is imperative that the ASCC give priority to evaluating and recommending to State and Federal governments the required numbers of occupational health and safety inspectors capable of enforcing any new national standards.

Second, the ASCC will need to address evidence presented to the "White" Senate Inquiry suggesting that toxic dust workplace exposure presents much greater health problems to the Australian community than is currently recognized. Community exposure through wind-borne dust and rainwater, for example, clearly has been insufficiently investigated.

Third, there is currently almost no credible research on the health impacts of nanoparticles despite increasing use of such technology in Australian industry. This data and regulatory gap must also be filled, thoroughly and competently and after broad consultations, by the ASCC.

Fourth, the ASCC needs to prioritise streamlining (and equity of access issues) for compensation processes linked with best scientific practice in the understanding of disease causation. Resolution of this issue will provide an important backdrop to implementation of existing and improved safety standards in the area of dust-related disease.
